# The Anatomical Correlation between the Internal Venous Vertebral System and the Cranial Venae Cavae in Rabbit

**DOI:** 10.1155/2013/204027

**Published:** 2013-11-21

**Authors:** David Mazensky, Eva Petrovova, Jan Danko

**Affiliations:** Department of Anatomy, Histology and Physiology, University of Veterinary Medicine and Pharmacy, Komenskeho 73, 041 81 Košice, Slovakia

## Abstract

The aim of this study was to describe the possible variations in the connection between the internal venous vertebral system and the cranial vena cava in rabbit using corrosion technique. The study was carried out on 40 adult New Zealand white rabbits. The venous system was injected by using Batson's corrosion casting kit number 17. We found the connection between the internal venous vertebral system and the cranial vena cava by means of the vertebral veins and the right azygos vein. The vertebral vein was present as independent tributary in 36 cases (90%). In the rest of the cases, it was found as being double, being triple, or forming a common trunk with other veins. The azygos vein was present as independent tributary of the cranial vena cava in 39 cases (97.5%). We found also a common trunk formed by the junction of the deep cervical vein, the right vertebral vein, and the azygos vein in one case (2.5%). The azygos vein received 6, 7, 8, or 9 pairs of dorsal intercostal veins. Documenting the anatomical variations in the rabbit will aid in the planning of future experimental studies and determining the clinical relevance on such studies.

## 1. Introduction

 The knowledge of anatomical variations is important for radiological and surgical procedures in humans and animals due to its practical and theoretical significance for experimental research and surgical practice in experimental and domestic animals [1].

 The variations of internal venous vertebral system and its main veins were best described in humans [2]. The internal venous vertebral system as venous plexus lying within the vertebral canal in the epidural space was described as a possible way of metastatic tumors means of injecting experiments in several animals [3]. Rabbits have been used as experimental model in many diseases [4]. 

 The aim of this study was to describe the possible variations in connection between the internal venous vertebral system and the cranial vena cava in rabbit using corrosion technique.

## 2. Material and Methods

The study was carried out on 40 adult rabbits (age 140 days). We used New Zealand white rabbits (breed HY+) of both sexes (female *n* = 20; male *n* = 20) with an average weight of 2.5–3 kg in an accredited experimental laboratory at the University of Veterinary Medicine and Pharmacy in Kosice. The animals were kept in cages under standard conditions (temperature 15–20°C, relative humidity 45%, and 12 h light period), and fed with granular feed mixture (O-10 NORM TYP). Drinking water was available for all animals *ad libitum*. The animals were injected intravenously with heparin (50,000 IU/kg) 30 min before they were sacrificed by intravenous injection of embutramide (T-61, 0.3 mL/kg). Immediately after killing, the vascular network was per fused with saline. Immediately after euthanasia, the vascular network was perfused with a physiological solution. The manual injection was done through the caudal vena cava. Batson's corrosion casting kit number 17 (Dione, České Budějovice, Czech Republic) in volume of 35 mL was used as a casting medium. The maceration was carried out in 2–4% KOH solution for a period of 8 days at 60–70°C. This study was carried under authority of decision number 2647/07-221/5.

## 3. Results

 We found the connection between the internal venous vertebral system and the cranial vena cava by means of the right and left vertebral vein and the right azygos vein.

**Figure 1:**
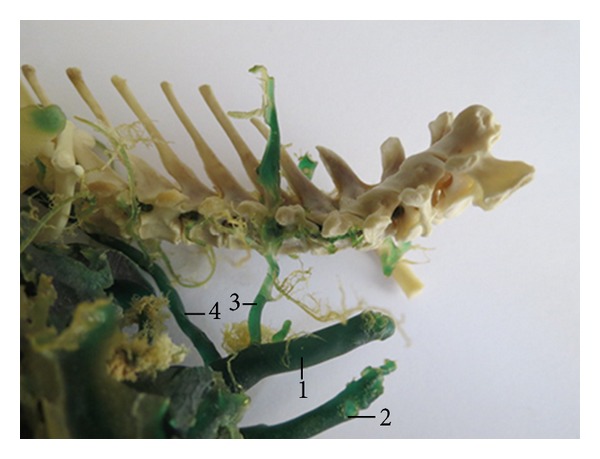
Vena (v.) vertebralis dextra as independent tributary of v. cava cranialis. (1) V. cava cranialis dextra, (2) v. cava cranialis sinistra, (3) v. vertebralis dextra, and (4) v. azygos dextra. Macroscopic image, dorsolateral view.

**Figure 2:**
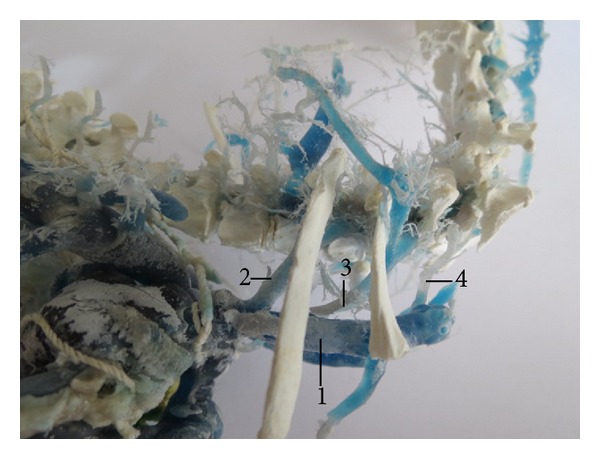
Triple v. vertebralis dextra. (1) V. cava cranialis dextra, (2) v. vertebralis dextra I, (3) v. vertebralis dextra II, and (4) v. vertebralis dextra III. Macroscopic image, ventrolateral view.

 The vertebral vein conveys the blood from the cervical and cranial thoracic region. As independent tributary the right vertebral vein opened into the cranial vena cava in 36 cases (90%; Figure 1). It was double in two cases (5%). In one case (2.5%), we found a common trunk formed by the right vertebral vein, the right deep cervical vein, and the right azygos vein. At the same corrosion cast, we found the second right vertebral vein as an independent tributary which was opened into the cranial vena cava. This vein received communicating branch coming from the first right vertebral vein and branch going out of the transverse canal of the cervical vertebrae. The right vertebral vein was triple in one case (2.5%; Figure 2). 

**Figure 3:**
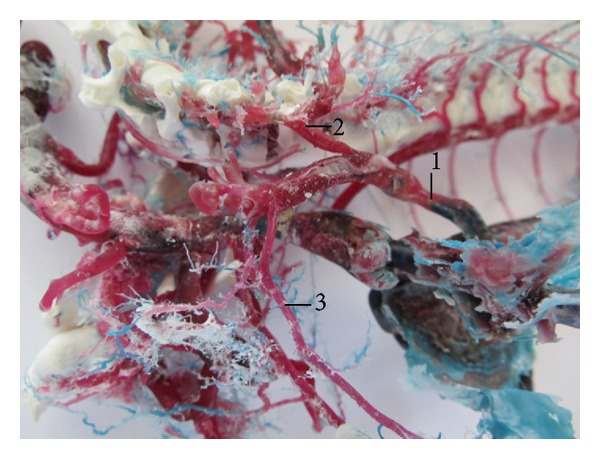
V. vertebralis sinistra as independent tributary of v. cava cranialis. (1) V. cava cranialis sinistra, (2) v. vertebralis sinistra, and (3) v. thoracica interna sinistra. Macroscopic image, dorsolateral view.

**Figure 4:**
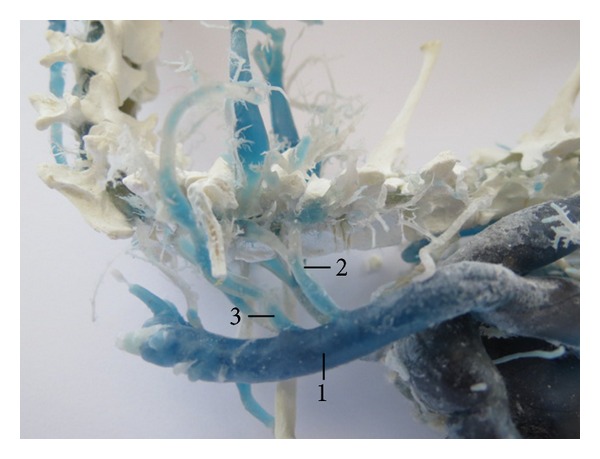
Double v. vertebralis sinistra. (1) V. cava cranialis sinistra, (2) v. vertebralis sinistra I, and (3) v. vertebralis sinistra II. Macroscopic image, lateral view.

The left vertebral vein as independent tributary of the cranial vena cava was found in 36 cases (90%; Figure 3). In 3 cases (7.5%), the left vertebral vein was present as double vein (Figure 4) and in one case (2.5%) as triple vein. In one case (2.5%), we found the left vertebral vein receiving two tributaries at the level of the head of the first rib.

**Figure 5:**
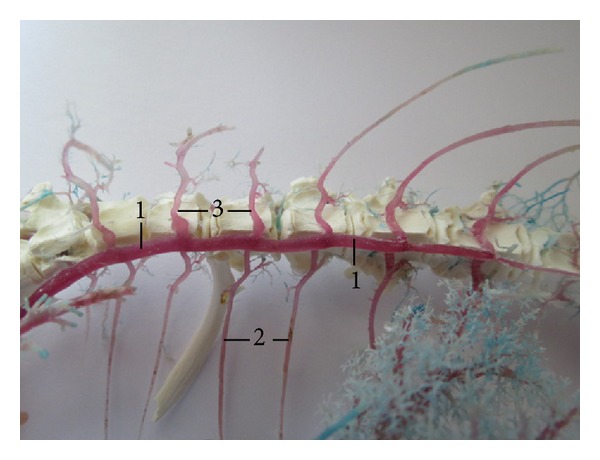
Vv. intercostales dorsales in number of 6 pairs. (1) V. azygos dextra, (2) vv. intercostales dorsales dextrae, and (3) vv. intercostales dorsales sinistrae. Macroscopic image, ventral view.

**Figure 6:**
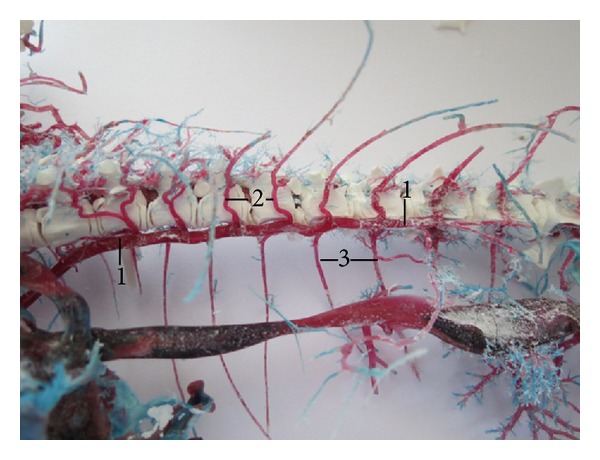
Vv. intercostales dorsales in number of 8 pairs. (1) V. azygos dextra, (2) vv. intercostales dorsales sinistrae, and (3) vv. intercostales dorsales dextrae. Macroscopic image, ventrolateral view.

 The right azygos vein starts its formation at the level of the first lumbar vertebrae by the junction of the first, second, and third pairs of lumbar veins. The vein entered the thoracic cavity ventrally to the vertebral column. Its thoracic segment received the dorsal intercostal veins in number of 6 pairs in 2 cases (5%; Figure 5), in number of 7 pairs in 10 cases (25%), in number of 8 pairs in 24 cases (60%; Figure 6), and in number of 9 pairs in 4 cases (10%). It emptied in the cranial vena cava in 39 cases (97.5%). The common trunk formed by the junction of right deep cervical vein, the right vertebral vein, and the right azygos vein was found as tributary of the cranial vena cava in one case (2.5%).

## 4. Discussion

 Till now, the vertebral vein was described as single independent tributary of the cranial vena cava [5, 6]. We found the vertebral veins as independent tributaries in 36 cases (90%). In the rest of the cases, it was found as being double, being triple, or forming a common trunk with other veins. Only one author described that the right vertebral vein was a tributary of the right costocervical vein [7]. Many works doubt the presence of the vertebral vein [8]. Krause [9] described the replacing of the vertebral vein by small vertebral branches coming out from the vertebral canal between the first, second, and third thoracic vertebras and emptying independently in the cranial vena cava. 

 The internal venous vertebral system is in direct connection with the vertebral veins. Metastatic abscesses and metastatic tumors can appear in locations that do not seem to be in line with direct spread from their primary focus. This type of spread is known as paradoxical metastasis. The internal venous vertebral system is today denoted as the way of paradoxical metastasis in patients with bone lesions with diagnosed carcinoma of the penis [3]. The clinical significance of the internal venous vertebral system is also obvious in patients with vein thrombosis of the thoracic limb [10].

The azygos vein was described as independent tributary of the cranial vena cava [5–9, 11]. The same situation was found in 39 cases (97.5%). We found also a common trunk formed by the junction of the deep cervical vein, the right vertebral vein, and the azygos vein in one case (2.5%). In all cases, the azygos vein started by the junction of bilateral first, second, and third pairs of lumbar veins. Craigie [8] described the point of arising at the level of junction of the first pair of lumbar veins. The azygos vein received 6, 7, 8, or 9 pairs of dorsal intercostal veins. Only in one study was the number of intercostal veins described [9]. In the present study, they were found in number of 7 on the right side and in number of 6 on the left side. 

The azygos vein is generally known as the bypass between the cranial and caudal vena cava. In different species of animals, the efferent venous system of adrenal glands was in direct connection with the internal venous vertebral system and by this way with azygos vein [12]. 

## 5. Conclusions

 It is important to report and document different anatomical variations of the azygos vein and the vertebral veins that may occur, because some anomalies of these veins can easily be confused with pathological conditions such as aneurysm, tumors, and enlarged lymph nodes. Documenting the anatomical variations in the connection between the internal venous vertebral system and the cranial vena cava in the rabbit and other species should be taken into account in imaging studies and surgical operations.
